# Modulation of Tumor-Associated Macrophages (TAM) Phenotype by Platelet-Activating Factor (PAF) Receptor

**DOI:** 10.1155/2017/5482768

**Published:** 2017-12-27

**Authors:** Ildefonso Alves da Silva Junior, Simone Cardozo Stone, Renata Marques Rossetti, Sonia Jancar, Ana Paula Lepique

**Affiliations:** Departamento de Imunologia, Instituto de Ciências Biomédicas, Universidade de São Paulo, São Paulo, SP, Brazil

## Abstract

Platelet-activating factor (PAF) plays an important role in the pathogenesis of several types of tumors. The biological effects of PAF are mediated by the PAF receptor (PAFR), which can be expressed by tumor cells and host cells that infiltrate the tumor microenvironment. In the present study, we investigated the role of PAFR expressed by leukocytes that infiltrate two types of tumors, one that expresses PAFR (TC-1 carcinoma) and another that does not express the receptor (B16F10 melanoma) implanted in mice that express the receptor or not (PAFR KO). It was found that both tumors grew significantly less in PAFR KO than in *wild-type* (WT) mice. Analysis of the leukocyte infiltration shown in PAFR KO increased the frequency of neutrophils (Gr1^+^) and of CD8^+^ lymphocytes in B16F10 tumors and of CD4^+^ lymphocytes in TC-1 tumors. PAFR KO also had a higher frequency of M1-like (CD11c^+^) and lower M2-like (CD206^+^) macrophages infiltrated in both tumors. This was confirmed in macrophages isolated from the tumors that showed higher iNOS, lower arginase activity, and lower IL10 expression in PAFR KO tumors than WT mice. These data suggest that in the tumor microenvironment, endogenous PAF-like activity molecules bind PAFR in macrophages which acquire an M2-like profile and this promotes tumor growth.

## 1. Introduction

Platelet-activating factor (PAF, 1-O-alkyl-2-acetyl-sn-glycero-3-phosphocholine) is an inflammatory lipid mediator produced through the activation of A_2_ phospholipase in response to different stimuli [1]. PAF is secreted by many different cell types, and the biological effects of this molecule are mediated by the activation of PAF receptor (PAFR), a G protein-coupled receptor expressed in monocytes/macrophages, polymorphonuclear leukocytes, platelets, endothelial cells, and other cell types as well as tumor cells [2–5].

Emerging evidence indicates that PAFR plays an important role in tumor growth [6–8]. Systemic treatment with PAFR antagonists resulted in the inhibition of tumor growth in murine melanoma, B16F10, and the human melanoma cell line, SK-MEL-37, engrafted in nude mice [9]. Transgenic mice overexpressing PAFR spontaneously developed melanocytic tumors [10]. In the tumor microenvironment, PAFR ligands can promote tumor growth, either by suppressing antitumor immune responses or by inducing tumor cell proliferation angiogenesis and production of growth factors [11, 12].

TAMs (tumor-associated macrophages) have been the subject of study for many research groups through the last few years. These are plastic cells that respond to the environment displaying a large phenotypic heterogeneity but that have been classified into two distinct extreme populations: classically activated macrophages (M1), which are characterized by high production of nitric oxide (NO) and reactive oxygen intermediates (ROI) and CD11c/IL-12 expression, and the alternatively activated macrophages (M2), identified by the expression of CD206 (mannose receptor) and IL-10, with high arginase activity and low NO production. In murine and human tumors, TAM generally exhibits an alternatively activated phenotype which is associated with the promotion of tumor growth, extracellular matrix remodeling, angiogenesis, and the suppression of adaptive immunity [13, 14].

Some tumor cells also express PAFR; upon activation of the receptor, intracellular programs are switched in tumor cells that promote their survival and proliferation [11, 15, 16]. We have recently shown that TC-1 carcinomas express PAFR and the addition of PAF increased tumor cell proliferation *in vitro*. Moreover, the addition of PAF to human carcinoma cells transfected with PAFR (KBP) increased cell proliferation, whereas in KBM cells, devoid of the receptor, PAF had no effect [17]. Human cancer cells derived from uterine adenocarcinoma (HEC-1A) have been shown to secrete PAF, and treatment with PAF receptor antagonists inhibited their proliferation [18]. There is also evidence that leukemia cell lines and cells derived from esophageal cancer express PAFR since the addition of PAF was able to stimulate transcription of the cyclooxygenase-2 enzyme, the activity of which was associated with tumor growth [19].

The tumor-promoting effect of PAF-like activity molecules generated in the tumor microenvironment can be dependent either on their effect on host cells or on tumor cells. The experiments that showed a reduction of tumor growth after *in vivo* treatment with PAFR antagonists do not allow to discriminate whether they blocked the receptor in host or tumor cells. Experiments by Sahu et al. [20] favor the first hypothesis. The authors showed that melanoma cells treated *in vitro* with PAF before implantation potentiated tumor growth in wild-type but not in PAFR KO mice.

In an attempt to understand the relative contribution of PAFR in the tumor microenvironment, we used two different tumor cell lines, B16F10 and TC-1 to inoculate wild-type mice (WT) or genetically deficient PAFR mice (PAFR KO). These tumor cells have different embryonic origins, generate subcutaneous tumors in 100% of the inoculated mice, and are very well characterized in the literature. Using these experimental models, we investigated tumor growth, tumor leukocyte infiltrate, and the TAM phenotype.

## 2. Methods

### 2.1. Cell Lines and Animals

The B16F10 melanoma cell lineage was purchased from the American Type Culture Collection (ATCC CRL­6475™, Manassas, VA, USA) and was maintained in DMEM (Dulbecco's Modified Eagle's Medium, GIBCO, Waltham, MA, USA) supplemented with 10% fetal calf serum (GIBCO), penicillin (100 units/mL), and streptomycin (100 *μ*g/mL). The TC-1 cell line was kindly donated by Dr. Wu (John Hopkins, Baltimore), this cell line is a murine carcinoma derived from lung epithelium, transduced with HPV16 E6/E7 and c-Ha-ras oncogenes [21]. TC-1 cell line was maintained in 10% FCS in RPMI supplemented with 400 *μ*g/mL neomycin. Cells have been regularly tested for mycoplasma and were free of this contamination. All cell cultures were incubated at 37°C under a humidified atmosphere of air containing 5% CO_2_.

C57BL/6 wild-type mice (WT, PAFR expressing; age 6–8 week) and age-matched PAFR-deficient (PAFR KO) mice on a C57BL/6 background, generated as described [22], were a kind gift of Professor Takao Shimizu (Department of Biochemistry, University of Tokyo). All mice were housed in the Department of Immunology's Animal Facility at the University of São Paulo. The animals were maintained in specific pathogen-free conditions, with 12 h light/dark cycles and water and chow ad libitum. All experimental procedures were performed following the guidelines adopted by the Brazilian College of Animal Experimentation (COCEA) and were approved by the Ethical Committee for Animal Research of the Institute of Biomedical Sciences of the University of São Paulo (protocol number 130/2015).

### 2.2. Mouse Tumor Model

Tumor cells lines (B16F10 or TC-1) were injected subcutaneously in the dorsal flank of C57BL/6 WT and PAFR KO mice as single cell suspensions (5 × 10^5^ in 100 *μ*L) in PBS^++^ (phosphate-buffered saline supplemented with 1 mM CaCl_2_, 0.5 mM MgCl_2_). Tumor formation and size were measured with a caliper until the 15th day. Mice were observed and measured with intervals of 2 or 3 days from the day when they were injected. Tumor volume was calculated using the equation: *V* = *D* ^∗^ *d* ^2^/2, where *V* is the tumor volume, *D* is the largest measured diameter, and *d* is the smallest measured diameter of the tumor.

### 2.3. Cell Suspension Preparations

All cell preparations were made using ice-cold 1x Hanks' solution with 15 mM HEPES, pH 7.4, 0.5 U/mL DNase I (Worthington Biochemical, Lakewood, NJ, USA) and 5% FBS. Tumors were harvested after mouse euthanasia. The tumor cell suspensions were obtained by the digestion of finely minced tissue with 1 mg/mL collagenase I and IV (Worthington Biochemical Corp., Lakewood, NJ) in the buffer described above in a ThermoMixer (Eppendorf, Germany) at 37°C for 45 min. Spleen-nucleated cell suspensions were obtained by tissue dissociation through a 70 *μ*m metal mesh and red cell lysis with ACK (ammonium-chloride-potassium) Lysis Buffer (Invitrogen, Invitrogen-Life Technologies, Carlsbad, CA, USA). Peritoneal macrophages were harvested after mouse euthanasia, by washing the peritoneal cavity with 5 mL ice-cold PBS. Cell viability, accessed by trypan blue staining, in the final suspensions was between 90% and 95%.

### 2.4. Flow Cytometry Analysis

Single cell suspensions were stained with different fluorochrome-conjugated antibodies (indicated in each figure). The antibodies used in this work were anti-CD4 (clone GK1.5), anti-CD8 (clone 53-6.7), antiGr1 (clone RB6-8C5), anti-CD11b (clone M1/70), anti-CD45 (clone 30-F11), and anti-F4/80 (clone BM8) purchased from BD Biosciences (San Diego, CA). Flow cytometry was performed in a FACSCanto II (BD Biosciences, San Jose, CA, USA), where 30,000–50,000 events were acquired. During data acquisition, debris and doublets were excluded. Data obtained were analyzed with the FlowJo software version 5.0 (TreeStar, Ashland, OR, USA).

### 2.5. Cell Sorting

CD45^+^ cells and leukocytes were sorted from total tumor suspensions by positive selection after incubation with biotin-conjugated anti-CD45 magnetic beads (Miltenyi Biotec, Germany) and loading in columns exposed to a magnetic field (MACS LS^+^ Separation Columns, Miltenyi Biotec). In general, we obtained 80–95% pure cells with at least 90% viability.

### 2.6. Quantification of Nitric Oxide, Arginase Activity, and IL-10

CD45^+^-sorted cells were seeded in 6-well culture plate (10^6^ cells/mL) in 10% RPMI treated with 10 ng/mL LPS (Sigma-Aldrich, St Louis, MO, USA) at 37°C for 72 h. Supernatants were then harvested for NO production and assessed by nitrite production in culture using the Griess reaction [23]. Arginase activity assay in total cell lysates was done as previously described [24] Aliquots of cell lysates were used for protein quantification by the Bradford assay (Bio-Rad; ref. 32). Murine IL-10 production was determined by ELISA (BD Biosciences, San Diego, CA, USA) according to the manufacturer's specifications.

### 2.7. Statistical Analyses

Tumor growth kinetics experiments were tested using the nonparametric Mann–Whitney *U* test. Data from all other experiments were tested by *t*-test, using the Prism 5.0 statistical program (GraphPad Software, San Diego, CA, USA). In all cases, *p* < 0.05 was considered significant. The number of animals or samples used in each experiment is indicated in the figure legends. Each experiment was repeated at least three times. Mostly, our data are represented as the average value of parameters obtained per experiment ± standard deviation (s.d.) unless otherwise indicated.

### 2.8. Data Availability

The datasets generated during and analyzed during the current study are available from the corresponding author on reasonable request.

## 3. Results

### 3.1. PAFR and Tumor Growth

**Figure 1:**
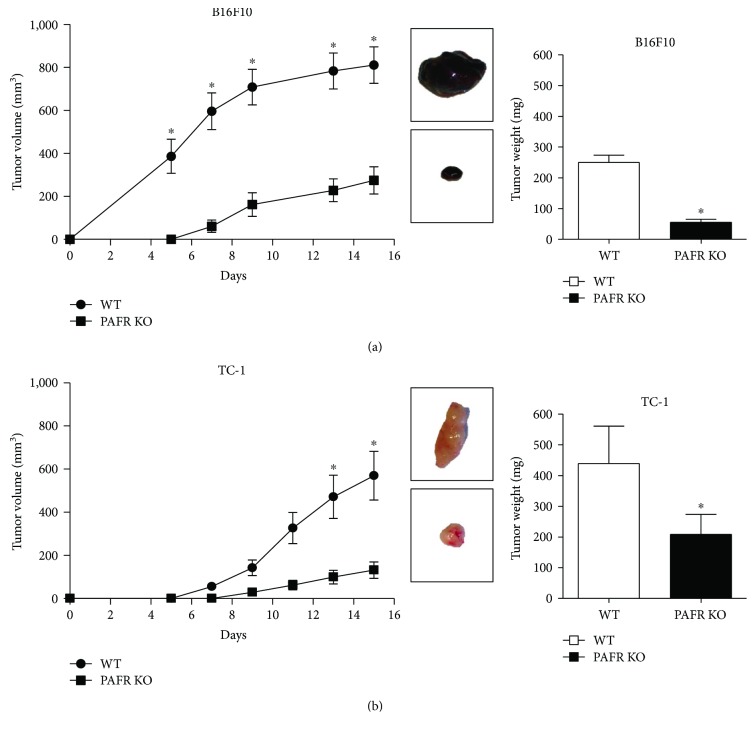
PAFR is important for B16F10 melanoma and TC-1 tumor growth. B16F10 melanoma cells and TC-1 tumor cells were injected (5 × 10^5^ cells) into the dorsal flank of C57BL/6 WT or PAFR KO mice. The left panel shows the tumor growth kinetics of B16F10 melanoma (a) and TC-1 (b). Representative macroscopic images of the tumors at 15 days postinoculation are shown to the right of the tumor growth kinetics; each tumor depicted in the images correspond to the tumors displayed in the adjacent curve. ∗ indicates *p* < 0.05 (Mann–Whitney *U* test). In the right panels, we show the average weight of the tumors at 15 days postinoculation. Data were obtained from 3 independent experiments with 4 animals per experimental group. ∗ indicates *p* < 0.05 (*t*-test).

We injected B16F10 melanoma and TC-1 carcinoma cells subcutaneously in WT and PAFR KO mice and followed tumor growth for 15 days. TC-1 cells express PAFR [17] whereas B16F10 do not [20]. After 6 days of cell inoculation, it was possible to detect palpable tumors in both mouse strains. However, in PAFR KO animals, the tumors were significantly smaller than in the WT. These results were consistently observed throughout the experiments for both, melanoma (Figure 1(a)) and for TC-1 carcinomas (Figure 1(b)). At the end of the experiment (day 15), tumor weight was also significantly smaller in PAFR KO mice. It is noteworthy that melanoma tumors had higher volume/weight rate (3.2) compared to TC-1 (1.2), which was compatible with the observation that melanoma was more edematous. Thus, the presence of PAFR in host cells seems to be relevant for tumor growth.

### 3.2. PAFR and Tumor Inflammatory Infiltrate

Our previous data suggest that PAFR signaling in the host plays a role in tumor growth. The tumor microenvironment is not only constituted by neoplastic cells but also several cell types recruited from the bloodstream, constituting the tumor inflammatory infiltrate. The inflammatory infiltrate can provide signals that inhibit or favor tumor growth [25]. Moreover, several of the cell types present in the tumor microenvironment express PAFR and can be modulated by its ligands. Therefore, we decided to investigate whether PAFR could control the tumor inflammatory infiltrate in our experimental models.

**Figure 2:**
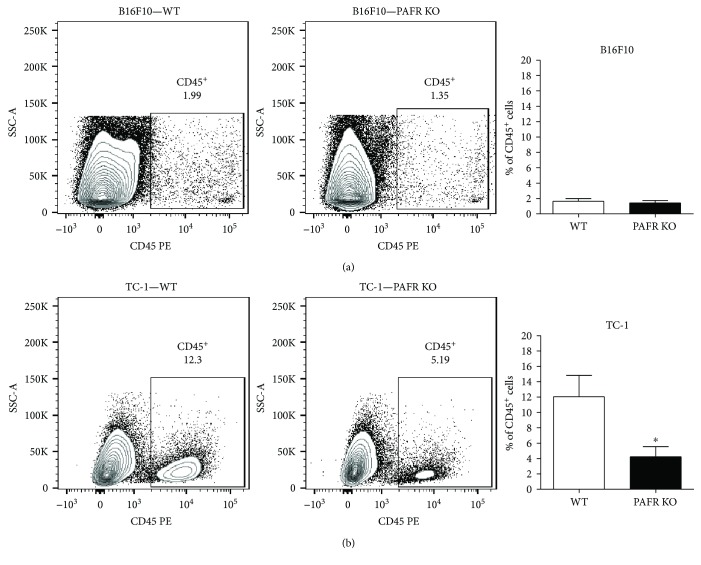
Inflammatory infiltrate in B16F10 melanoma and TC-1 tumors. Single cell suspensions of TC-1 (a) or B16F10 (b) tumors from WT and PAFR KO animals injected 15 days earlier were analyzed by flow cytometry. Cells were labeled with anti-CD45 antibody and 50,000 events were acquired per sample, using a FACSCanto cytometer. In the left panel: dot plots depicting SSC-A X CD45 expression (panleukocyte marker) of tumors from WT and PAFR KO animals. These plots were obtained by previously gating out debris and doublets. The gates indicate the CD45^+^ populations. In the right panels, we show the average percentage of CD45^+^ cells in each experimental group. Data were obtained from 3 independent experiments with 4 animals per experimental group. ∗ indicates *p* < 0.05.

We harvested and digested tumors from wild-type and PAFR KO mice 15 days after tumor cells inoculation and analyzed the inflammatory infiltrate by flow cytometry. In melanoma tumors, the tumor inflammatory infiltrate (CD45^+^ cells) corresponded to 1.6 ± 0.3% of the total cells and there was no difference in the percentage of CD45^+^ cells between the two groups of animals (WT versus PAFR KO; Figure 2(a)). In TC-1 tumors, however, the inflammatory infiltrate was almost 10 times higher than in B16F10 melanoma and a significant reduction in the number of infiltrated cells was observed in PAFR KO (Figure 2(b)).

**Figure 3:**
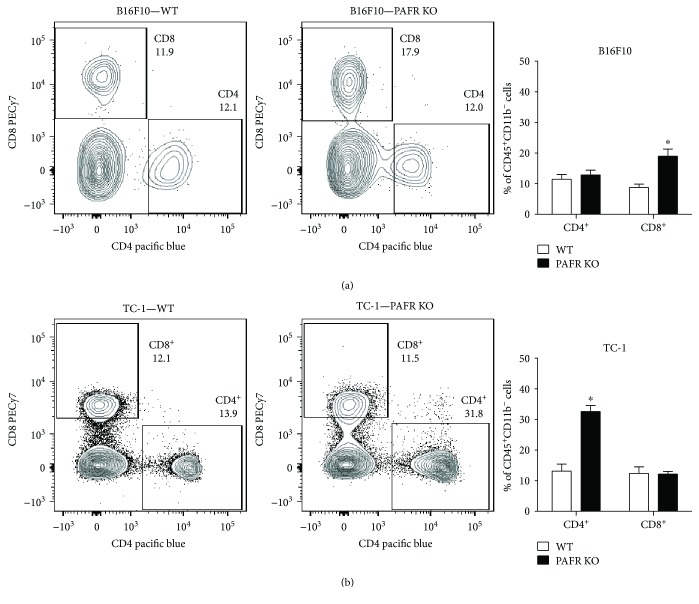
Characterization of lymphoid tumor infiltrate. Total single cell suspensions of B16F10 (a) or TC-1 (b) tumor from WT and PAFR KO animals were analyzed by flow cytometry (50,000 events). The left panels show a representative analysis of the lymphocyte (CD4^+^ and CD8^+^) populations. The CD4 and CD8 populations were analyzed within the CD45^+^CD11b^−^ gate, after gating out debris and doublets. The panels to the right show the quantification of these experiments, represented as the average percentage of cells expressing CD4 or CD8, within the CD45^+^CD11b^−^ population. Data were obtained from 3 independent experiments with 4 animals per experimental group. ∗ indicates *p* < 0.05.

Next, we evaluated the frequency of lymphocyte populations in the tumors. We observed a twofold increase in the frequency of CD8^+^ T cells, within the CD45^+^ population in PAFR KO animals injected with melanoma when compared to WT animals (Figure 3(a)). In contrast, in TC-1 tumors, it was the CD4^+^ cell population that was increased in PAFR KO (Figure 3(b)).

**Figure 4:**
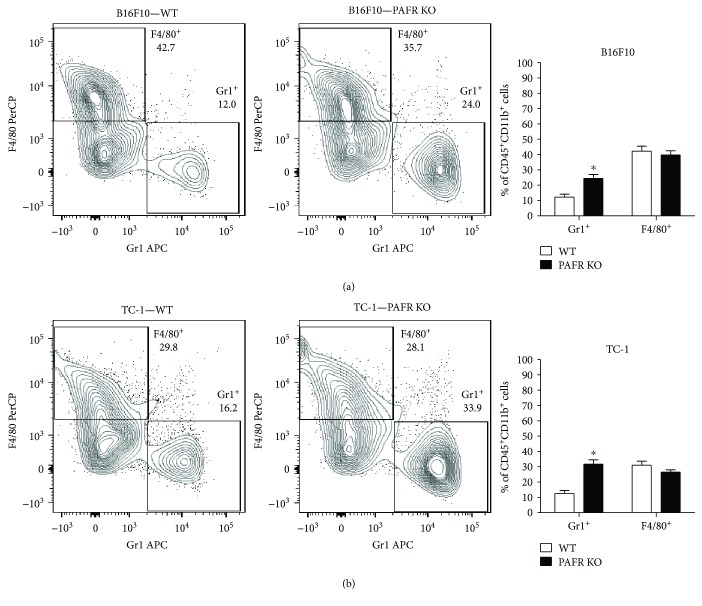
Characterization of myeloid tumor infiltrate. Total single cell suspensions of B16F10 (a) or TC-1 (b) tumors from WT and PAFR KO animals were labeled with antibodies against the indicated markers plus anti-CD45 and anti-CD11b and analyzed in a FACSCanto, where 50,000 events were acquired. In the left panels, we show a representative analysis of the myeloid populations from 3 independent experiments with 4 animals per group. The cells were analyzed within the CD45^+^CD11b^+^ gate, after exclusion of debris and doublets. In the right panel, we show the mean percentage of the frequency of Gr1^+^ and F4/80^+^ cells within the CD45^+^CD11b^+^ population. ^∗^ *p* < 0.05.

Regarding the myeloid populations (macrophages and neutrophils) that were recruited to the tumors, we found that B16F10 melanoma recruited more macrophages (F4/80^+^ cells) than TC-1 tumors; macrophages corresponded to 42 ± 3% of the CD45^+^CD11b^+^ population whereas in TC-1 tumors, macrophages corresponded to 28.7 ± 2.6% of this population (Figure 4). Interestingly, in both tumor models, the frequency of neutrophils (CD45^+^CD11b^+^Gr1^+^) was significantly higher in PAFR KO mice than in WT mice (Figures 4(a) and 4(b)). Together, these results indicate that the absence of PAFR in the host cell determines the recruitment of different leukocyte populations into the tumor stroma.

### 3.3. PAFR and Tumor-Associated Macrophages (TAM) Phenotype

We have previously shown that the activation of PAFR reprogram mice and human macrophages towards an anti-inflammatory phenotype [26]. We therefore decided to investigate the phenotype of TAM in our experimental models. Although these cells are highly heterogeneous, they are classified into two extreme subtypes: the classically activated M1, which has a proinflammatory profile and expresses CD11c as a phenotypic marker, and the alternatively activated macrophages M2, which exhibit an anti-inflammatory profile and express CD206 [27]. Thus, we determined the frequency of CD11c and CD206 cells within the CD45^+^CD11b^+^F4/80^+^ macrophage population.

**Figure 5:**
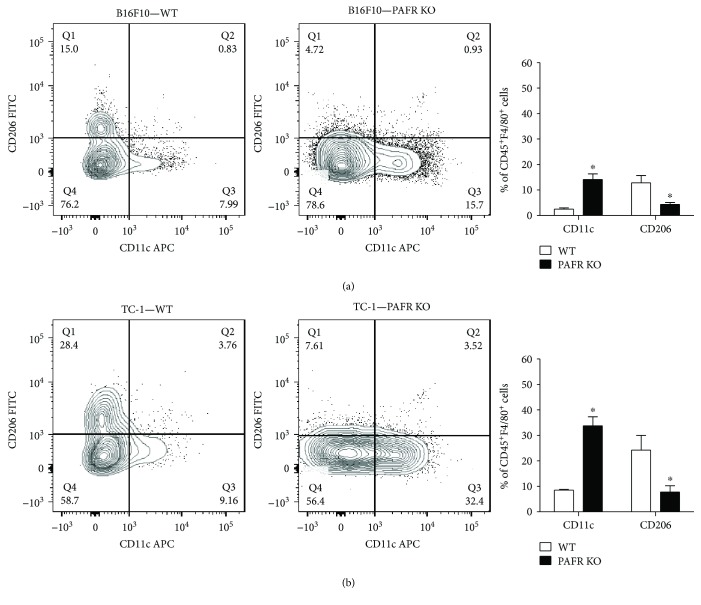
PAFR-modulated TAM phenotype. In a (B16F10) and b (TC-1), we show a representative analysis of the macrophage populations (CD45^+^CD11b^+^ F4/80^+^) expressing the M1-like marker, CD11c, or the M2-like marker, CD206, from WT and PAFR KO tumors. ∗ indicates *p* < 0.05 for WT compared with PAFR KO tumors. Each experimental group contained 4 animals.

Figure 5 shows that PAFR KO mice had a significantly higher frequency of TAM expressing the CD11c (M1-like) and a lower frequency of cells expressing CD206 (M2-like) molecule in both melanoma (Figure 5(a)) and TC-1 (Figure 5(b)) tumors when compared to the WT groups of each strain.

**Figure 6:**
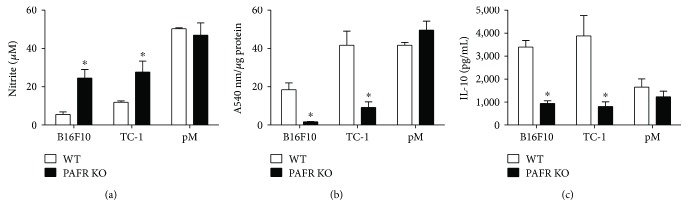
TAMs from PAFR KO tumors show the M1-like phenotype. CD45^+^ cells were purified from tumor suspensions from animals by positive selection using CD45-coated magnetic beads (Miltenyi Biotec) and stimulated with 10 ng/mL of LPS. After 72 hours of incubation at 37°C in 10% SFB RPMI, the culture supernatants were harvested for nitric oxide (nitrite) production detection by Griess reaction (a) and IL-10 detection by ELISA (c). The cellular extracts were used for determination of arginase activity (b) normalized by the total protein concentration measured by BCA kit. ∗ indicates *p* < 0.05 for WT compared with PAFR KO tumors. Each experimental group contained 4 animals. pM are peritoneal macrophages treated in exactly the same way as tumor infiltrating cells, used as control for NO detection and arginase activity assays.

This was confirmed in macrophages (CD45^+^ cells) isolated from the tumors and stimulated with 10 ng LPS for 72 hours in culture. We observed that macrophages from PAFR KO animals produced significantly higher concentration of nitrite (Figure 6(a)), indicative of iNOS activity, and had lower arginase activity (Figure 6(b)) than cells from WT animals. There was also lower concentration of IL-10 in the supernatant of cultures of leukocytes from tumors from PAFR KO mice (Figure 6(c)). Thus, in the WT mice, the TAMs are predominantly M2 whereas in PAFR KO mice, the TAMs are predominantly M1. It can be suggested that during tumor growth, the generation of PAFR ligands by activating the receptor reprograms the macrophages towards the M2 phenotype which favors tumor growth. In the absence of PAFR, the activated M1 macrophages are able to control tumor growth.

## 4. Discussion

In the present study, we showed that the growth of B16F10 melanoma and TC-1 carcinoma is reduced in mice lacking the PAF receptor when we compared to WT mice. Our results indicate that during the growth of melanoma B16F10 and TC-1 carcinoma PAF receptor ligands are produced in the tumor microenvironment and control the recruitment and phenotype of inflammatory cells to the tumor, promoting the accumulation of M2 macrophages and stimulating tumor growth. Our observations are made even more robust in light of the different PAFR status of B16F10 and TC-1 cell lines. TC-1 cells express PAFR [17], whereas B16F10 do not [20], which indicates that PAFR signaling in the tumor cells was not important in our experimental context.

Evidence from a previous work showed that PAFR has an important role in tumor growth based on studies employing selective antagonists of PAFR. Blockade of PAFR with the antagonist, WEB2170, reduced the growth of Ehrlich ascites tumor (EAT) [28] and melanoma B16F10 growth in C57BL/6 mice [29]. PAF receptor antagonist ginkgolide B inhibits tumorigenesis and angiogenesis in colitis-associated cancer [30]. Several tumor cells express PAFR and its activation is involved in tumor cell survival and proliferation. In the present work, we showed that the protumoral effect of PAF ligands was due to the modulation of TAM phenotype rather than on tumor cell proliferation.

Interestingly, while we observed that deficiency in PAFR expression caused a reduction in total leukocyte recruitment (CD45^+^ population), this effect was directed to specific populations, as within the CD45^+^ infiltrate, neutrophils and T cells still had higher frequency in tumors from PAFR-deficient mice than in tumors from WT mice, suggesting that PAFR mediated the recruitment of monocytes/macrophages. Our results also indicate that during tumor growth, chemokines that promote leukocytes migration to the tumor are produced in response of PAFR activation inside the tumor microenvironment. Previous work has shown that in PAFR KO animals with Ehrlich tumor ascites, elevated levels of CXCL2 and CCL2 chemokines controlled the recruitment of myeloid cells to the tumor [31].

In our study, the increased frequency of neutrophils and intratumoral CD4^+^/CD8^+^ lymphocytes correlated with the inhibition of tumor growth, suggesting that PAFR may be involved in the recruitment of these cells into the tumor stroma. Although we did not investigate the phenotype of these lymphocytes specifically, our data suggest that in PAFR KO mice, these cells might have an antitumor function. Indeed, when macrophages were depleted from TC-1 tumors, not only was an increase in the T cell tumor infiltration found but also the presence of antitumor-specific CD8 cells in the infiltrate [32].

PAF appears to have a pivotal role in macrophage function. Macrophages undergo functional and phenotypic changes in response to signals from the tumor microenvironment. The role played by macrophages in the biology of neoplasias is complex, because macrophages may assume anti- or protumor phenotype [27, 33–35]. M1 phenotype or “classically activated” macrophages have antitumor activity and macrophages of the M2 phenotype are considered protumor [36–38]. In our study, we found no significant differences in the frequency of macrophages that infiltrate melanoma and TC-1 tumors when comparing PAFR KO and WT animals. However, when we analyzed the activation profile of these cells, we observed that in the PAFR KO animals there was a significant shift in the frequency of macrophages from M1 to M2 phenotype. Previous work from our group showed that during EAT growth, macrophages in the ascites presented morphology of nonactivated macrophages and after treatment *in vivo* with PAFR antagonists (BN52021 or SRI63441), the macrophages acquired an activated morphology, and this was accompanied by a significant reduction in EAT growth [28, 39]. The clearance of apoptotic cells by macrophages requires the scavenger receptor CD36 and PAFR and induces macrophage reprogramming towards the M2 phenotype [26]. De Oliveira et al. [29] showed that PAFR antagonist decreased the phagocytosis of apoptotic cells by macrophages and inhibit the production of anti-inflammatory cytokines and mediators. These results suggest that during tumor growth, the clearance of apoptotic cells by TAM as well as the generation of PAF or PAF-like activity molecules in the tumor microenvironment modulates the macrophages into the M2 suppressor phenotype.

Solid tumors can display systemic effects on leukocyte populations, modulating the immune response even before cells reach the tumor microenvironment or promoting the proliferation of protumoral cells, such as myeloid-derived suppressor cells [40]. This is an important aspect to be considered during tumor growth since molecules produced in the tumor microenvironment can circulate and signal to lymphoid organs, increasing hematopoiesis, leading to accumulation, mainly of myeloid cells, in secondary lymphoid organs [41, 42]. We have previously shown that myeloid cells accumulate in the spleen of TC-1 tumor-bearing mice [32]. Here, we also observed that mice bearing tumors have larger spleens and significantly higher cellularity than those without tumors. Interestingly, this tumor-associated splenomegaly did not occur in PAFR KO mice (Supplementary Figure S1). Whether this was a direct effect on cell proliferation and survival in lymphoid organs or a result of the diminished tumor growth, with concomitant reduction in the secretion of molecules that could stimulate leukocytosis, is yet to be investigated. Either way, it seems that the activation of PAFR-dependent pathways can interfere with the balance of leukocyte populations in the spleen and thus potentially modulate the adaptive immune responses to the tumor.

Together with the data presented in this manuscript, we can assume that part of this mechanism may relate to the signaling through PAFR. Interestingly, STAT3 upregulation is a hallmark of many cancer models, not only in cancer cells but also in the inflammatory infiltrate [43]. For instance, cervical carcinoma cells can induce the tolerogenic phenotype in macrophages through the secretion of IL-6 and PGE2 [44]. PAF/PAFR can activate the IL-6/STAT3 axis contributing to the epithelial-mesenchymal transition in nonsmall lung cancer cells [45]. Therefore, the idea that PAFR signaling may have direct and indirect effects in promoting cancer progression and growth seems consistent.

Our results clearly show that PAFR ligands modulate inflammatory cells in the tumor microenvironment, mainly macrophages, promoting protumoral effects, through the induction of the M2 macrophage phenotype.

## Supplementary Materials

Supplementary Materials Figure S1: PAFR controls spleen cellularity in tumor bearing animals. Number of nucleated single cells in suspensions from spleens of WT (white bars) or PAFR KO (black bars) mice bearing B16F10 or TC-1 and animals without tumors (naïve) were determined using a hemocytometer. Results are expressed as the average of cell numbers of spleens of 4 mice per experimental group (*n* = 3). ∗ indicates *p* < 0.05 for WT compared with PAFR KO tumors.
