# Supplementary material for: Modulation of Tumor-Associated Macrophages (TAM) Phenotype by Platelet-Activating Factor (PAF) Receptor
Source: J Immunol Res. 2017 Dec 27;2017:5482768. doi: 10.1155/2017/5482768 (PMC5763242; doi:10.1155/2017/5482768)

**Figure S1. PAFR controls spleen cellularity in tumor bearing animals**. Number of nucleated single cells in suspensions from spleens of WT (white bars) or PAFR KO (black bars) mice bearing B16F10 or TC-1 and animals without tumors (naïve) were determined using a hemocytometer. Results are expressed as the average of cell numbers of spleens of 4 mice per experimental group (n=3). * indicates p< 0.05 for WT compared with PAFR KO tumors.


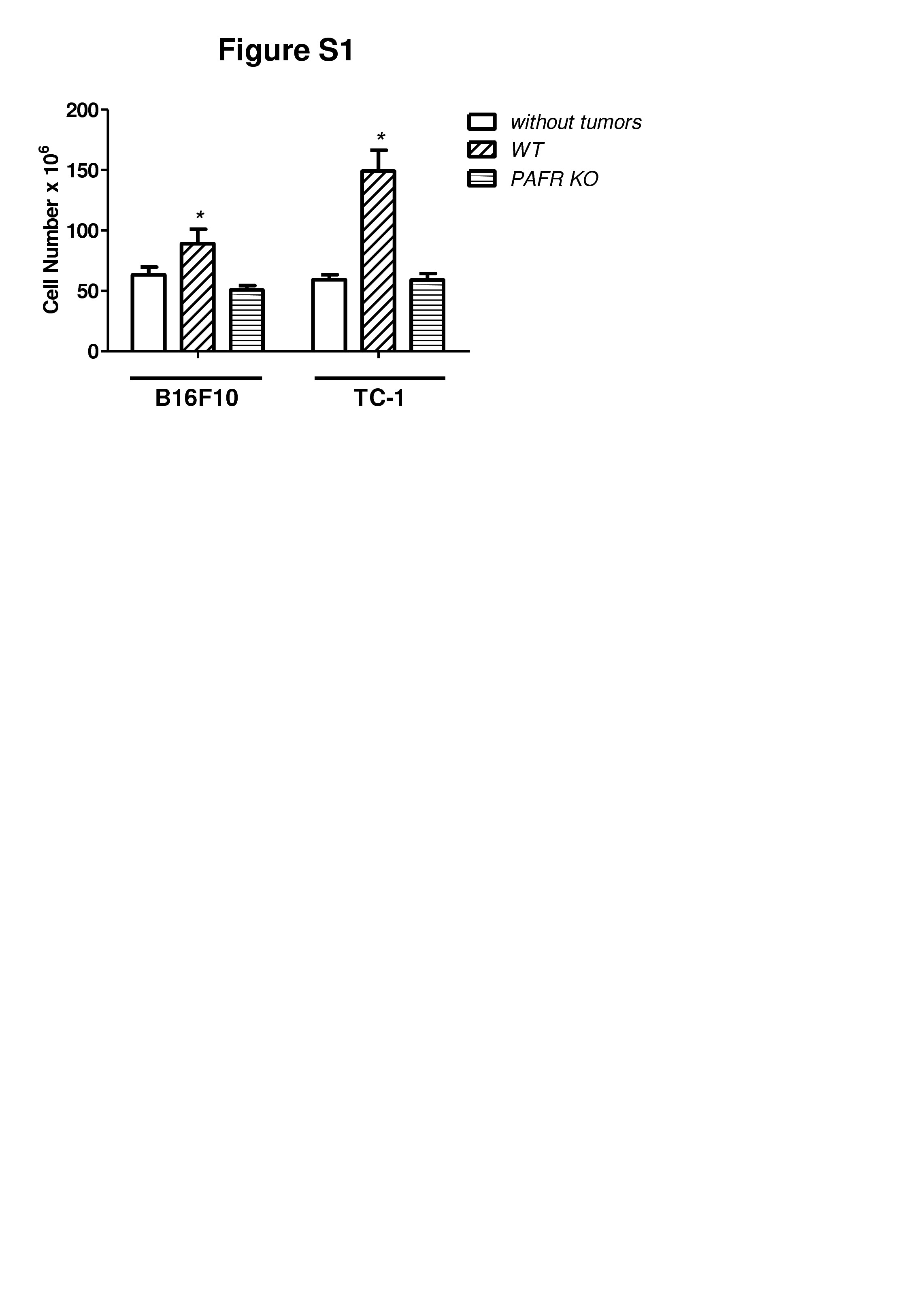

Supplement: Supplementary Materials — Figure S1: PAFR controls spleen cellularity in tumor bearing animals. Number of nucleated single cells in suspensions from spleens of WT (white bars) or PAFR KO (black bars) mice bearing B16F10 or TC-1 and animals without tumors (naïve) were determined using a hemocytometer. Results are expressed as the average of cell numbers of spleens of 4 mice per experimental group (n = 3). ∗ indicates p < 0.05 for WT compared with PAFR KO tumors. [file 5482768.f1.docx]
